# HOXA10 and HOXA11 Methylation: Epigenetic Barriers to Endometrial Receptivity in ART

**DOI:** 10.3390/genes16101230

**Published:** 2025-10-17

**Authors:** Dmitry Kudlay, Vsevolod Kiselev, Gennady Sukhikh

**Affiliations:** 1 Department of Pharmacology, Institute of Pharmacy, I.M. Sechenov First Moscow State Medical University (Sechenov University), st. Trubetskaya, 8, Building 2, Moscow 119991, Russia; 2 Laboratory of Personalized Medicine and Molecular Immunology, National Research Center—Institute of Immunology Federal Medical-Biological Agency of Russia, Kashirskoe Highway, 24, Moscow 115522, Russia; 3 Department of Pharmacognosy and Industrial Pharmacy, Faculty of Fundamental Medicine, Lomonosov Moscow State University, Leninskie Gory, 1, Moscow 119991, Russia; 4 Epinenetics Laboratory at the National Medical Research Center for Obstetrics, Gynecology and Perinatology Named After Academician V. I. Kulakov of the Ministry of Healthcare of Russia, Oparina Street, 4, Moscow 117997, Russia; vkis10@mail.ru; 5 National Medical Research Center for Obstetrics, Gynecology and Perinatology Named after Academician V.I. Kulakov of the Ministry of Healthcare of Russia, Oparina Street, 4, Moscow 117997, Russia; g_sukhikh@oparina4.ru

**Keywords:** endometrial receptivity, IVF, HOXA10, HOXA11, epigenetic dysregulation, gene demethylation, epigallocatechin-3-gallate, indole-3-carbinol

## Abstract

The global prevalence of infertility has reached critical levels, making it one of the most pressing issues in modern society. Assisted reproductive technologies (ARTs), particularly in vitro fertilization (IVF), are the primary treatment methods for infertility. However, even under optimal conditions, the pregnancy rate per IVF cycle does not exceed 40%, while the live birth rate remains around 30%. A key unresolved challenge in ART is impaired endometrial receptivity (ER), which significantly contributes to repeated implantation failure (RIF). Advances in molecular and genetic diagnostics have revealed that gynecological conditions associated with infertility, such as chronic endometritis, uterine fibroids, polycystic ovary syndrome (PCOS), and tuboperitoneal factor infertility, are often linked to epigenetic alterations. Specifically, abnormal hypermethylation of the promoter regions of the HOXA10 and HOXA11 genes has been observed in women of reproductive age with these conditions. Such epigenetic dysregulation negatively impacts ER and is associated with infertility. The methylation status of HOXA10 and HOXA11 may serve as a potential diagnostic marker for evaluating and treating infertility. These markers can be assessed using available molecular genetic techniques, including real-time PCR. A promising therapeutic approach to improve ER involves the use of epigallocatechin-3-gallate and indole-3-carbinol, which have been shown to demethylate and restore the expression of HOXA10 and HOXA11. Epigenetic regulation holds significant potential for enhancing the effectiveness of ART programs, offering new avenues for addressing infertility and improving reproductive outcomes. This review consolidates the current body of knowledge regarding the epigenetic regulation of endometrial receptivity. It outlines fundamental scientific data on epigenetic mechanisms and discusses contemporary diagnostic and pharmacological intervention strategies.

## 1. Successes and Failures of Assisted Reproductive Technology

Infertility remains a significant medical and social issue, affecting 12.6–17.5% of reproductive-aged couples worldwide. In Russia, the incidence of infertile marriages reaches 17.2–24%, exceeding the critical demographic threshold of 15% [1,2,3,4,5,6,7,8]. The primary treatment method is Assisted Reproductive Technology (ART), which involves procedures where conception and early embryo development stages occur outside the body [4]. The most common ART procedure is in vitro fertilization (IVF). The increasing average age of first-time mothers, now over 30 in developed countries, has further driven the demand for ART, including IVF [9,10,11,12,13,14].

Despite over 40 years of successful IVF practice and the availability of detailed clinical guidelines, the problem of treatment inefficacy remains highly relevant [14,15,16]. Live birth rates have plateaued, showing no recent progress [17]. The reasons for failure are multifactorial, including chromosomal abnormalities, sperm DNA damage, suboptimal embryo development, and impaired endometrial receptivity [18,19]. A particular challenge is Recurrent Implantation Failure (RIF), defined as the failure to achieve pregnancy after multiple IVF cycles with the transfer of good-quality embryos [15,16,17,18,19,20,21].

Retrospective studies indicate that clinical pregnancy rates consistently decline from 52% in the first IVF cycle to 28% in the third, with an estimated RIF prevalence of 15% [22]. Successful implantation requires synchronized interaction between a viable embryo and a receptive endometrium [18,19,20]. However, even under optimal conditions, the probability of pregnancy per single IVF cycle does not exceed 30–40%, while the live birth rate is only 25–30% [18,21,23,24], highlighting the need for further research to improve treatment outcomes.

## 2. Endometrial Receptivity: Genetics and Epigenetics

The secretory phase of the menstrual cycle is studied most because, in this phase, the endometrium acquires a receptive phenotype, allowing blastocyst implantation. Endometrial receptivity (ER) refers to the ability of the endometrium to interact with an embryo. It is a complex of structural and functional characteristics with clear temporal and spatial parameters that determine the ability of the endometrium to implant an embryo. The period of receptivity is known as the window of implantation, WOI [25]. In the normal endometrium, progesterone and estrogen signaling act in synergy to inhibit epithelial proliferation and facilitate the transition to a receptive state during WOI [26]. WOI is registered from day 19 to day 20 of the cycle and lasts for 4–5 days [20]. According to other authors, WOI lasts from day 19 to day 24 of a 28-day cycle [27]. If no implantation occurs, the levels of progesterone and estrogen decrease, which is accompanied by constriction of the spiral arteries, and the endometrium sheds; then, the cycle starts over [18]. However, the optimal WOI lasts less than 48 h, with great inter-individual variability [28]. Abnormal ER, including WOI shifts and pathological lesions, have been observed in many patients with RIFs [27].

Next-generation sequencing (NGS) has revolutionized genome research. Many genome sequencing projects that previously took years using Sanger sequencing methods can now be completed in a few hours using NGS [29]. Currently, several different NGS platforms using different sequencing technologies are available. These technologies open up new opportunities for molecular diagnostics, including in molecular studies of ER [30].

Using cutting-edge technology, it has been demonstrated that thousands of coding genes change their level of expression in the endometrium throughout the menstrual cycle [31]. The HGEx-ERdb database contains data on the expression status of 19,285 genes at different stages of the menstrual cycle and in various types of therapy, including hormonal, pregnancy, use of contraceptives, and various conditions. The database provides information about the molecular characteristics of genes or encoded proteins, e.g., promoter sequence, amino acid sequence, location, and molecular function of the encoded protein [32].

Homeobox genes of the *HOX* class are leading candidates for regulating endometrial differentiation when it is being prepared for embryonic implantation [33]. Homeobox genes encode transcription factors required for embryonic morphogenesis and cell differentiation [34]. These genes contain a common sequence element of 183 nucleotides; mammals are known to have 39 *HOX* genes [35,36]. The HOXA10 and HOXA11 genes and the proteins they encode are currently considered to be one of the key regulators of ER that determine fertility in general [37,38]. The most important role of the HOXA10 and HOXA11 genes is to control the expression of progesterone receptors in the endometrium and ensure its function [38].

The expression of HOXA10 and HOXA11 varies depending on the phase of the menstrual cycle. HOXA10 and HOXA11 are expressed in the proliferative phase of the endometrium, and their expression increases during the secretory phase. The most dramatic increase in their expression occurs during implantation. If implantation is successful, the early pregnancy decidua maintains high expression of HOXA10 and HOXA11 mRNA [39,40,41].

**Figure 1:**
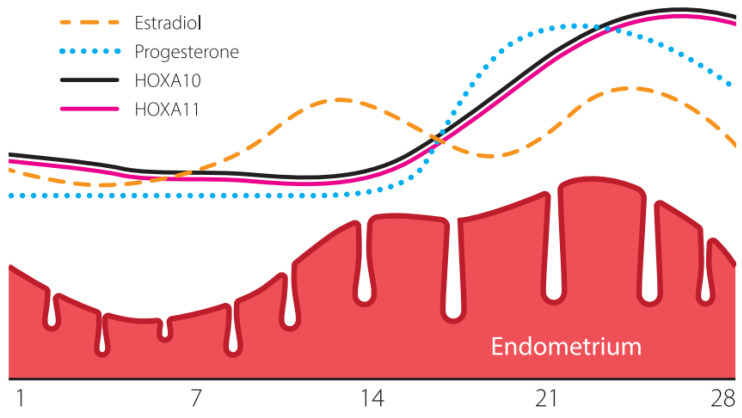
Change pattern in the expression of the HOXA10 and HOXA11 genes in the human endometrium over the menstrual cycle.

Figure 1 shows the expression pattern of the HOXA10 and HOXA11 genes in the human endometrium over the menstrual cycle. The HOXA10 and HOXA11 genes are key transcription moderators. In women with normal menstrual cycles, there is a surge in HOXA10 and HOXA11 expression during the mid-secretory phase. On the contrary, a decline in HOXA10 and HOXA11 expression in the secretory phase results in a low rate of embryo implantation. ER disorders have been studied in several gynecological diseases leading to infertility, including endometriosis, polycystic ovary syndrome, uterine fibroids, and hydrosalpinx. The expression of HOXA10 and HOXA11 is reduced in women with each of these disorders compared to the control subjects. Changes in the expression of HOXA genes cause infertility in humans, primarily due to defects in endometrial receptivity and impaired implantation [39,40].

Several studies have assessed the role of the HOXA10 and HOXA11 genes in WOI occurrence [42,43]. They are now characterized as regulators that have pleiotropic effects on many aspects of endometrial development, including stromal decidualization, leukocyte infiltration, and the development of pinopodes [35,36,39,42].

Epigenetic factors lead to a change in gene expression without affecting the DNA sequence; however, they may be inherited [44,45]. Due to its significantly greater plasticity, the epigenotype, rather than the genotype, is assumed to be the main inducer of infertilityn [46]. Epigenetics encompasses several different phenomena, such as DNA methylation, histone modifications, RNA interference, and genomic imprinting [47]. These epigenetic mechanisms induce the expression of genes associated with the regulation of transcription, the growth of endometrial epithelium, angiogenesis, and the proliferation of stromal cells during the proliferative phase. During the secretory phase, epigenetic mechanisms promote gene expression associated with hormonal response, signal transmission, decidualization, and embryo implantation [45]. In vitro studies and examination of specific cell types have shown that epithelial and stromal cells undergo certain epigenetic changes that modify their transcription networks to perform their functions during decidualization and implantation [45,48].

**Figure 2:**
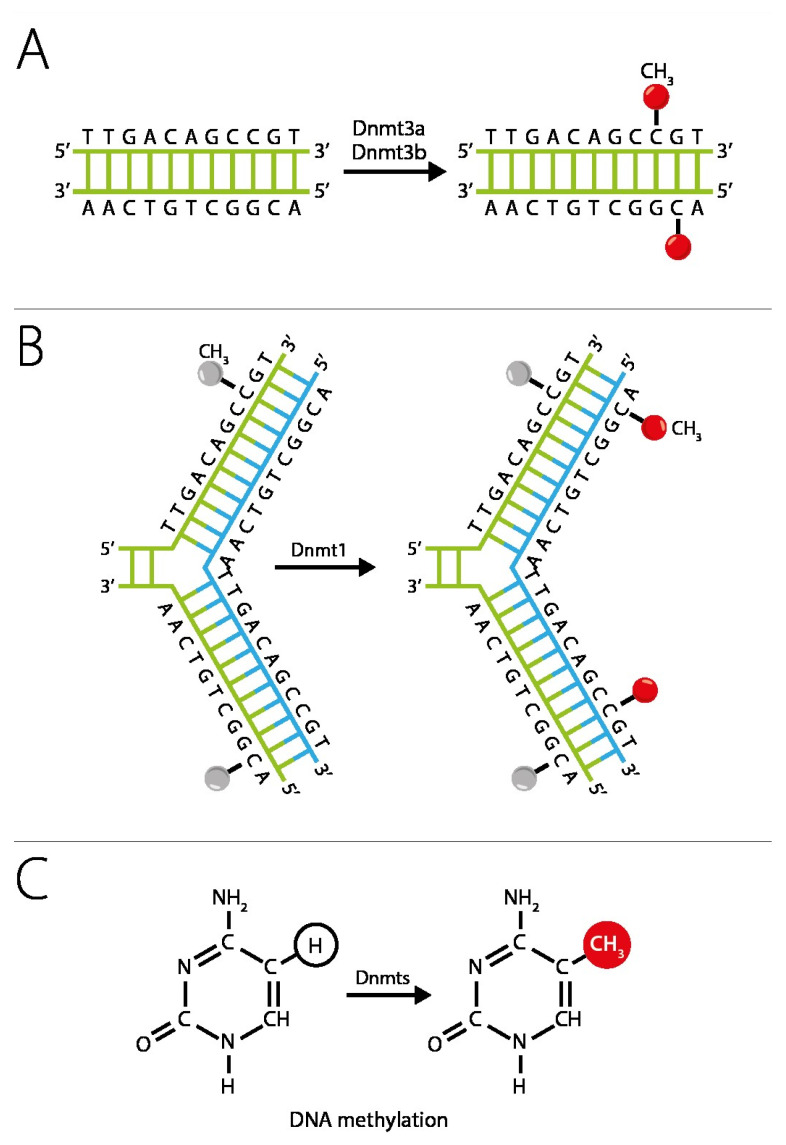
DNA methylation. (**A**) Dnmt3a and Dnmt3b are DNA methyltransferases (DNMTs) that carry methyl groups (red) to the bare DNA de novo. (**B**) Dnmt1 maintains DNA methylation patterns during replication. When DNA undergoes semiconservative replication, the parental DNA retains the original DNA methylation pattern (gray). Dnmt1 binds at replication foci and closely reproduces the original DNA methylation pattern, adding methyl groups (red) to the newly formed daughter strand (blue). (**C**) The DNA methyltransferase (Dnmts) family catalyzes the transfer of the methyl group to the fifth carbon of the cytosine residue to form 5-methylcytosine (5mC).

One of the key epigenetic mechanisms that control cyclic changes in the endometrium is DNA methylation. The DNA methylation profile correlates with its biological age, and changes in this profile are associated with endometrial dysfunction [31,45,49]. Changes in DNA methylation rate during the menstrual cycle are tissue-specific since the methylation in the endometrial tissue varies with the cycle phase; in contrast, such changes are not observed in blood samples. DNA methylation is a chemical modification of DNA in which a methyl group is transferred to the fifth carbon atom of cytosine to form 5-methylcytosine (5mC). DNA methylation is catalyzed by DNA methyltransferases (DNMT) [31,50]. Figure 2 shows the scheme of DNA methylation: DNA methyltransferases (Dnmts1, Dnmt3a Dnmt3b) catalyze the transfer of the methyl group from S-adenylmethionine (SAM) to the fifth carbon atom of the cytosine residue to form 5-methylcytosine (5mC). Methylation occurs mainly in genome sites where cytosine is proximal to guanine (CpG dinucleotides) [51,52].

There is every reason to believe that abnormal promoter hypermethylation of the HOXA10 and HOXA11 genes causes their functional shutdown, which in turn leads to disruption of the implantation process and eventually to infertility [38,42,53,54].

## 3. Endometrial Receptivity Disorders as a Reason for Implantation Failure

Today, one of the primary reasons for implantation failure is poor ER, which is one of the leading causes of RIFs [19]. Poor ER is considered one of the main reasons for non-pregnancy if a high-quality embryo has been transferred in IVF procedure [55]. Several studies have suggested that up to two-thirds of implantation failures are associated with ER defects, whereas poor quality of the embryo accounts for only one-third of IVF cycles failures [56,57].

ER disorders have been studied in several gynecological diseases associated with infertility, such as endometriosis, polycystic ovary syndrome, and uterine fibroids. It is noted that such disorders of endometrial development that prevent implantation and lead to female infertility are associated with a decrease in the expression of HOXA10 and HOXA11 genes as a result of their hypermethylation [39,40,58].

In a study by Andersson K.L. et al. (2014) it was found that patients with endometriosis do not demonstrate cyclic changes in the expression of HOXA10/HOXA11 or their activation during the window of implantation, which may partly explain the infertility associated with this disease. Further studies to evaluate the expression pattern of HOXA10/HOXA11 allowed for the presentation of DNA methylation as a possible mechanism of altered gene expression in endometriosis [58].

In patients with endometriosis, the implantation rate decreases both during natural cycles and during ART, even in patients with minimal manifestations of the disease [59]. The HOXA10 and HOXA11 genes regulating in-uterine embryogenesis and the regulation of ER are involved in the pathogenesis of infertility associated with endometriosis. As we have previously noted, the expression of HOXA10 and HOXA11 increases dramatically during the WOI and remains elevated throughout the secretory phase (Figure 1). However, patients with endometriosis do not demonstrate such an increase [60,61]. A study by Naqvi et al. showed that the expression of HOXA10 was suppressed in endometriosis and that the methylation of the HOXA10 gene differed by 1.3 times. Epigenetic dysregulation of the *HOX* gene expression in endometriosis leads to persistent changes in the ER [2,40].

Polycystic ovary syndrome (PCOS) is a complex endocrine disorder caused by both genetic and epigenetic factors [62]. Infertility associated with PCOS is a consequence of chronic oligoanovulation [63]. Even with the correction of ovulatory disorders, the pregnancy rate in PCOS remains low, and the rate of spontaneous abortion is high. In women with PCOS, 30% to 50% of all conceptions end in miscarriage. Several studies have found that even with ovulation induction in PCOS, changes in the ER lead to infertility [64]. Endometrial biopsy obtained from women with PCOS in ovulatory cycles showed that HOXA10 expression during the secretory phase is reduced in PCOS compared to healthy fertile women [40,65].

Uterine fibroids are the most common benign tumor of the uterus in women of reproductive age. Estrogen and progesterone are known to stimulate fibroid growth. This is supported by their receptor levels. Fibroids have much higher estrogen receptors than the uterine lining and muscle. They have even higher levels of progesterone receptors [66]. Clinical symptoms of fibroids are abnormal uterine bleeding, painful and heavy menstruation, infertility, and miscarriage. Fibroids are diagnosed in 5–10% of women with infertility [67]. Several studies have found that HOXA10 is expressed in human myometrium and that its expression depends on the phase of the menstrual cycle. Endometrial expression of HOXA10 and HOXA11 is significantly reduced in uteri with submucosal fibroids compared to healthy fertile women. This effect is not only localized to the endometrium overlying the fibroid; decreased HOXA10 expression is observed throughout the endometrium. This effect of fibroids on the endometrium suggests the presence of a diffuse factor that may influence ER away from the fibroid. It is hypothesized that fibroids alter ER by secreting transforming growth factor beta (TGFβ) and thus decreasing the expression of HOXA10, which is required for implantation [40,67].

## 4. Epigenetic Silencing of Receptivity Regulating Genes

**Figure 3:**
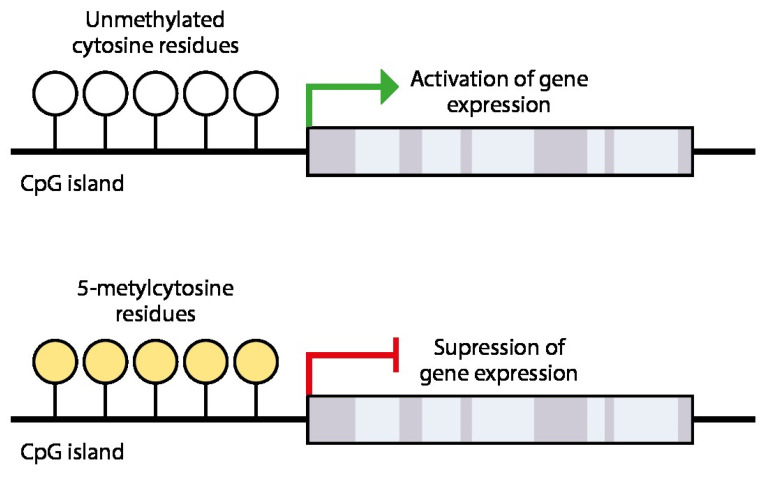
Mechanism of epigenetic gene silencing. Methylation of cytosine residues in the CpG island located in the gene promoter region. When CpG islands are methylated (yellow), gene expression is suppressed, and the gene becomes silent. CpG-island (C: Cytosine; p: Phosphate; G: guanine).

DNA methylation is a cellular memory mechanism and carries important information about gene expression programming [51]. In the vast majority of cases, cytosines that are part of CpG dinucleotides are subject to methylation. About 80% of CpG dinucleotides are scattered throughout the genome, but 20% are collected in clusters known as CpG islands [68]. In normal somatic cells, most CpG islands are unmethylated. When DNA is methylated in gene promoters, it may prevent the transcription mechanism from accessing the DNA and initiating transcription [51] (Figure 3). CpG methylation blocks transcription factors from binding to gene promoters. In vitro studies have shown that even the methylation of a single CpG dinucleotide at a transcription factor binding site can inhibit transcription factor binding [51]. When promoter CpG islands are methylated, the associated gene is usually silenced due to suppression of transcriptional activity [41].

Transcription factors are proteins that recognize specific DNA sequences to initiate DNA transcription. CpG islands are targets for transcription factors that activate gene expression. Unmethylated CpG islands are open for transcription and gene expression. However, when CpG islands are methylated, transcription factors are unable to bind to the site, resulting in suppressed gene expression [69].

Methylation of promoters inhibits their recognition by transcription factors and RNA polymerase because methylated cytosines preferentially bind to a protein known as methyl–CpG binding protein (MeCP). When a promoter region normally recognized by an activating transcription factor is methylated, its transcription is inhibited [70].

Of note, the Ten Eleven Translocation (TET) family proteins—TET1, TET2, and TET3—were identified relatively recently, in 2009–2011. These enzymes catalyze the sequential oxidation of 5-methylcytosine to 5-hydroxymethylcytosine, 5-formylcytosine, and 5-carboxylcytosine, which are intermediates in its conversion back to unmodified cytosine [71]. These proteins are Fe^2+^- and O^2−^ dependent dioxygenases that exhibit marked tissue specificity [72].

TET proteins, which play a key role in DNA demethylation, are now recognized as critical epigenetic regulators essential for maintaining cellular identity and genomic stability. Their dysregulation or mutation leads to abnormal hypermethylation and inactivation of tumor suppressor genes and is associated with the development of a wide range of cancers [72,73]. According to Szczepanska et al., decreased TET1 mRNA expression in the eutopic endometrium of infertile women with endometriosis during the mid-secretory phase is a potential mechanism for hypermethylation of the HOXA10 gene [74].

Thus, it has become clear that DNA methylation is a reversible process, the reversal of which can occur not only through passive mechanisms (via inhibition of DNMT enzymes—epigenetic “writers”) but also through active mechanisms involving TET proteins, which act as “epigenetic erasers” that remove methyl groups from cytosine residues in DNA. Furthermore, DNMT and TET enzymes balance each other [75]. For a long time, DNA methylation was considered a stable epigenetic event that suppresses gene expression and is faithfully transmitted to daughter cells only during DNA replication [71].

## 5. Molecular Genetic Methods for Assessing Endometrial Receptivity

The human endometrial transcriptome has been extensively studied over the past decade to search for diagnostic markers of receptivity and gain insight into the complex regulation of endometrial function [76].

Currently, various methods can be used to assess ER: histology, electron microscopy, immunohistochemistry, examination of the gene expression profile in endometrial biopsy samples, etc. All of these methods are aimed to identifying the changes in the endometrium that occur during the WOI period (formation of pinopodes, appearance of structural proteins on the apical surface of epithelial cells, synthesis of cytokines, growth factors, adhesion molecules, etc.) [23,76].

A meta-analysis by Craciunas L. et al. (2019) included 163 non-interventional studies (88,834 women) that assessed potential ER markers [77]. Most of the studies included were focused on markers of ER in the context of IVF (138/163; 85%) and ultrasound markers (120/163; 74%). The studies were based in 36 countries. This meta-analysis identified the following markers of endometrial receptivity:

1. Ultrasound markers: endometrial thickness; trilinear pattern; endometrial volume; uterine artery pulsatility index; resistance parameters in the uterine, arcuate, radial, basal, and spiral arteries—assessed on the day of the ovulation trigger injection and the day of embryo transfer;
2. Markers in endometrium biopsy samples and the method of evaluation:
  - BLC6 (B-cell lymphoma 6 protein)—immunocytohistochemistry [78];
  - Inhibin A—quantitative polymerase chain reaction (PCR) [79];
  - Integrins αvβ3, α4 β1, α1β1—immunocytohistochemistry [80];
  - Ligand of L-selectin—immunohistochemistry and Western blotting [81];
  - Aromatase P450—quantitative PCR [82];
  - Vascular endothelial growth factor (VEGF)—immunocytohistochemical study [83];
  - Human chorionic gonadotrophin/luteinizing hormone receptor (HCG/LH-R)—streptavidin-biotin-peroxidase complex technique for immunohistochemicstry [84];
  - Macrophage colony-stimulating factor (M-CSF) Macrophage colony-stimulating factor M-CSF—streptavidin-biotin-peroxidase complex technique for immunohistochemistry [84];
  - HOXA10—streptavidin-biotin-peroxidase complex technique for immunohistochemistry [84];
  - Endometrial receptivity array (ERA)—gene expression profile [24].
3. Hysteroscopy: ring type of gland arrangement and the presence of well-developed varicose-like vessels; assessment of endometrial blood flow (>29 mL/min/100 g) [77];
4. Pinopods count by electron microscopy (minimum 60 fields at ×2000 magnification) [77].

In their study, He A. et al. (2023) conducted a non-invasive RNA-sequencing-based endometrial receptivity test (nirsERT, rsERT) by analyzing the transcriptomic profile of 144 uterine fluid samples with three different receptivity statuses from 48 patients participating in IVF protocols. A retrospective observation of a small cohort showed that 77.8% of IVF patients with a predicted normal WOI had a successful intrauterine pregnancy, while none of the 3 patients with a displaced WOI had a successful pregnancy. The nirsERT technique can predict the period of WOI relatively accurately and serves as a non-invasive method for assessing ER [27].

In recent years, several personalized transfer studies have been conducted on patients with RIF. A group of researchers developed a transcriptional signature that based on an algorithm for integral assessment of gene expression in endometrial biopsies, allows them to assess the endometrial receptivity to embryo implantation (personal WOI). The ERA^®^ (Endometrial Receptivity Array) test, registered by Igenomix in 2009, is a molecular genetic test designed to evaluate ER. Considering that approximately one-third of infertile women may suffer from WOI shift, ERA has become a promising tool in ART programs [78]. A systematic review with meta-analysis by Liu Z. et al. (2022) included 11 publications on the results of studies in infertile women who underwent ERA and subsequent protocols of IVF/ICSI. It was found that infertile women with a good prognosis did not have a statistically significant improvement in embryo transfer outcomes, but the rate of pregnancy increased significantly in infertile women with RIFs of endometrial origin [85].

Advances in technology have enabled the microarray approach to emerge as a new tool for the evaluation of ER. A genomic, proteomic, and lipidomic evaluation of ER means a comprehensive assessment of endometrial genes, lipids, and proteins. Assessing receptivity using this three-step approach may provide insight into potential markers of implantation. To date, genomic analysis is limited because not every gene change affects protein expression. Lipidomic analysis has recently gained popularity because lipids are strictly monitored during the implantation period. Thus, with recent advances in microarray technology, genomic, lipidomic, and proteomic analyses of ER may provide tools and criteria for evaluating ER in the near future [21].

Knyazeva E.A. et al. (2019) conducted full-genome transcriptomic profiling using Affymetrix microarrays of endometrial samples from 15 women with tuboperitoneal factor infertility and a history of repeated IVF failures. When planning the study, potential genes were selected that could affect the IVF outcomes, after which the expression of these genes was analyzed by PCR to construct classifiers of IVF outcomes based on the expression of pairs and triplets of genes. A total of 47 samples were analyzed, including 15 from women who became pregnant and 32 from women in whom no pregnancy occurred. Of the 235 triplets, only one triplet consisting of the MSX1 (HOX7), HOXA11, and TP53I3 genes exceeded the sensitivity and specificity threshold. The study revealed that the MSX1, HOXA11, and TP53I3 triplet allows for predicting the outcomes of the ART program in women with a history of multiple IVF failures and tuboperitoneal factor infertility. The use of a classifier based on the MSX1 (HOX7), HOXA11, and TP53I3 triplet can determine endometrial receptivity and create an individual prediction of the IVF outcome in women with a history of tuboperitoneal infertility and RIF [23].

In their study, Nazarenko T.A. et al. (2019) examined the methylation status of the promoter regions of the HOXA10 and HOXA11 genes in endometrial biopsy samples from 34 women with tubal factor infertility. All study subjects had a history of at least two embryo implantation failures. The association between HOXA10 and HOXA11 promoter methylation levels, clinical parameters, and implantation outcomes after IVF cycles in women with RIF and tubal infertility was examined. Endometrial samples were collected during the WOI period before the IVF cycle to assess the methylation status of the HOXA10 and HOXA11 promoters using bisulfite conversion and quantitative real-time PCR. It was found that methylation of the HOXA10 promoter region was observed in 76.5% (26/34) patients and of HOXA11 in 100.0% (34/34) patients. The findings suggest that methylation status may be a significant predictor of implantation success during IVF cycles [42].

In their study, Sukhikh G.T et al. (2015) studied the methylation status of the promoter regions of the HOXA10 and HOXA11 genes in endometrial biopsy samples of 25 patients aged 28 to 40 years suffering from CE-related infertility [38]. After the genomic DNA isolation, 25 test samples were obtained, which were then subjected to bisulfite conversion. For subsequent PCR analysis and sequencing, fragments I and II of the promoter regions of the HOXA10 and HOXA11 genes, respectively, were selected since, according to the literature, it is in these fragments that the highest level of methylation is observed compared to healthy fertile women [61,86]. It was found that methylation in the promoter region of the HOXA10 gene was observed in 21 out of 25 patients, that is, in 84% of cases, and the HOXA11 gene was observed in 16 of 25 patients, or in 64% of cases. According to the literature data, the endometrium of healthy women of reproductive age demonstrates no promoter methylation of these genes [61,86]. Thus, we can conclude that women with CE-related infertility have abnormal promoter hypermethylation of the HOXA10 and HOXA11 genes, leading to a decrease in their expression and a decrease in the level of the proteins they encode [38].

HOXA10 gene methylation was analyzed. It was compared to patient medical histories. Specifically, it was compared to the duration of infertility from CE. A clear correlation was found between the two. To illustrate, the average level of the HOXA10 gene methylation was 5.7%, 29%, and 41% for infertility lasting from 6 months to 1 year, for 2–3 years, and for 5–6 years, respectively, while patients with long-term (10 years or more) infertility and/or recurrent pregnancy loss had methylation levels approaching 50% [38].

The results obtained are consistent with previously described data: the fact of abnormal hypermethylation and, as a consequence, inactivation of HOXA genes is detected in the vast majority of infertile women with endometriosis, uterine fibroids, and idiopathic infertility [41,60,86].

From the above analysis, it follows that the level of methylation of HOXA10 and HOXA11 may be an informative predictor of embryo implantation, whereas real-time PCR with preliminary bisulfite conversion may be an inexpensive and accessible method for assessing the methylation status of genes (Table 1).

Currently, among all commercially available tests for assessing endometrial receptivity, the most extensively studied and widely used in clinical practice is the Endometrial Receptivity Array (ERA), first introduced to the scientific community in 2011 [87]. The ERA is a DNA microarray designed to identify the transcriptomic profile of 238 genes with differential expression during the transition from the pre-receptive to the receptive endometrial status. This test determines the optimal time for embryo transfer into the uterus (WOI) and improves the chances of a successful pregnancy in IVF, particularly in cases of RIF [88].

Data from more than 10 retrospective clinical studies (only some of which utilized a control group undergoing standard IVF protocols) suggest that the ERA test may be beneficial for both RIF patients and the general population. However, prospective studies and the only randomized clinical trial conducted to date have demonstrated the efficacy of ERA primarily for RIF patients [89]. Furthermore, a recent systematic review by Mei et al., analyzed only studies that originally reported reproductive outcomes of patients undergoing ERA-guided euploid embryo transfer (EET) to eliminate the interference of embryo quality. It revealed that ERA could not optimize reproductive outcomes in EET cycles, neither in the general infertile population nor in patients with a history of previous failed embryo transfers [90].

In addition to the limited and contradictory validated data supporting the efficacy of ERA in the general population and among RIF patients, other disadvantages of this test include its high cost and the necessity for an additional embryo transfer cycle and biopsy procedure.

With the advancement of omics and bioinformatics technologies (genomics, transcriptomics, proteomics, metabolomics), the number and diversity of markers for assessing molecular and transcriptomic changes in the endometrium during WOI are rapidly growing [91,92].

A promising predictive tool for assessing endometrial receptivity is the RNA-seq-based endometrial receptivity test (rsERT), proposed by He et al. in 2021. It utilizes a combination of transcriptomic analysis of a panel of biomarker genes via RNA sequencing (next-generation high-throughput sequencing) and machine learning [93].

Subsequent studies have shown that applying the rsERT test to uterine fluid analysis in RIF patients undergoing IVF allows for highly accurate prediction of their WOI, enables personalized embryo transfer, and significantly improves pregnancy outcomes with fewer cycles [27,94,95].

The rsERT test is non-invasive, which is a clear advantage over the ERA biopsy. It uses a simple uterine fluid sample, reducing patient discomfort. On the downside, the test is still costly, requires more research for validation, and needs better methods to collect samples and prevent RNA degradation (Table 1) [27,94].

An important direction in modern research and development aimed at creating innovative diagnostic/prognostic tests and targeted therapies is epigenetics, or, in the modern language of multi-omics technologies, epigenomics. Epigenetics is referred to as the “second informational code of life”. In addition to the deterministic genetic code, it provides a powerful resource for complex, multi-level regulation of vital processes and the maintenance of cellular identity in multicellular organisms. Disruption of epigenetic regulation leads to abnormal changes in the gene expression profile and creates a molecular-genetic basis for disease development. To date, the determining role of epigenetics in the etiology of most common diseases has been proven. Modern epigenetics is a vast scientific industry offering new, previously inaccessible opportunities for diagnosis and treatment. The reversibility, and hence the regulatability, of epigenetic modifications makes epimarkers extremely attractive therapeutic targets for the development of next-generation targeted drugs. Scientists rightly call the 21st century the century of epigenetics. They predict that deciphering the epigenetic mechanisms of development and life processes will lead to a real revolution in biology and medicine.

The paramount role here is assigned to DNA methylation—the dominant and most studied mechanism of epigenetic regulation of gene expression. With properly selected marker genes critical for pathogenesis, a test based on DNA methylation can diagnose pathology and assess predisposition to its development with high sensitivity and specificity.

Conducting DNA methylation-based tests allows for rapid screening of a large number of biological samples, does not require significant financial costs, and can be performed using the widely available PCR method in its various modifications, including methylation-specific RT-PCR [96].

Currently, commercially available in vitro diagnostic tests based on biomarkers exist for colorectal cancer, glioblastoma, hepatocellular carcinoma, lung and bladder cancer, as well as cervical and prostate cancer [97,98,99].

In recent years, studies on DNA methylation of candidate genes and the entire genome (methylome) in various pathologies, including in the endometrium of RIF patients undergoing IVF, have been actively conducted [100,101].

Convincing evidence has been obtained for the key role of HOXA10 and HOXA11 genes in regulating endometrial receptivity and the pathogenesis of RIF [89,102,103]. Their potential use as markers in diagnostic/prognostic tools and targets for targeted therapy in infertile patients has been demonstrated [42]. However, further research is needed for full validation and standardization of such epigenetic diagnostics.

Due to ethical concerns associated with using in vivo approaches to study human embryo implantation, interest in using three-dimensional in vitro models (3D cell/tissue culture models and organoid models) to study the physiological processes of endometrium-trophoblast interaction during implantation and post-implantation development has been growing over the past decade [104].

Owing to the rapid progress in molecular biology and multi-omics technologies, new epigenetic markers of endometrial receptivity are being identified, particularly genes regulating embryo adhesion, immune tolerance, and pregnancy maintenance (LIF, HOXA10, ITGB3, SERPINE1, SERPINE2, TAGLN2), as well as non-coding RNAs (lncRNA H19, LUCAT1, miR-let-7, miR-495-3p), enabling non-invasive diagnosis in blood serum [50,91,105,106].

The general trend in the development of diagnostic and prognostic tools for determining endometrial receptivity status and the personalized window of implantation (WOI) involves the creation of highly accurate, non-invasive (performed on biological fluids—blood serum, uterine fluid, cervical mucus) biological mechanism-based molecular tests (including those operating at the single-cell level) utilizing modern “omics” (genomics, epigenomics, transcriptomics, proteomics, lipidomics, metabolomics, microbiomics) approaches. Concurrently, it will be necessary to address challenges related to their technical standardization, dynamic monitoring, and clinical validation.The use of new diagnostic and therapeutic strategies is expected not only to enhance the effectiveness of ART, particularly IVF, but also to improve the overall diagnosis and treatment of infertility, as well as to refine endometrial-targeted contraceptive methods [91,107,108].

## 6. Methods for Improving Endometrial Receptivity

Several authors describe endometrial scratching as a non-pharmacological method for enhancing the implantation properties of the endometrium before an IVF program. This is a local injury to the endometrium performed during hysteroscopy or using a pipette biopsy curette [85,109]. It is assumed that local injury to the endometrium triggers a local inflammatory response, which may contribute to the interaction between the blastocyst and the endometrium. The inflammatory response induces the production of proinflammatory cytokines and the subsequent recruitment of macrophages and other immune cells involved in the implantation process [85,109]. Krasnopolskaya et al. established a tendency toward an increase in pregnancy rate after scratching in patients with extremely thin endometrium [110]. However, currently, there are conflicting opinions about the effectiveness of endometrial scratching, and this technique requires further study [85,109]. Some authors suggest that endometrial damage contributes to a decline in the number of implantations [27].

In addition, the literature describes the transvaginal administration of granulocyte colony-stimulating factor (G-CSF) to enhance the implantation properties of the endometrium [111,112]. The proposed mechanism of action is based on G-CSF involvement in endometrial vascular remodeling, local immune modulation, and cellular adhesion pathways. However, this mechanism remains poorly understood. Li J. conducted a meta-analysis of studies that assessed the effect of transvaginal G-CSF on pregnancy rates and endometrial thickness in women undergoing IVF procedures and found that G-CSF was associated with a higher clinical pregnancy rate compared to placebo, but the increase in endometrial thickness was not statistically significant [111].

Currently, epigenetic silencing of progesterone-responsive genes, including HOXA genes, caused by their promoter hypermethylation is considered a mechanism for the development of progesterone resistance in endometriosis, which is proposed to be treated with DNA methylation inhibitors to restore the activity of the silent genes and, as a consequence, improve endometrial receptivity [41]. One of the promising areas for pharmacological improvement of endometrial receptivity is the demethylation of the HOXA10 and HOXA11 genes [37].

The nucleoside inhibitor 5-Aza-2′-deoxycytidine (5′-AZA) inhibits DNA methyltransferase and increases HOXA10 expression if its promoter region is hypermethylated. Wang L et al. studied the effect of 5′-AZA on embryo implantation in the Jeg-3 endometrial spheroid cells. The percentage of methylated CpG islands in the HOXA10 promoter region was 72.0% in the group without 5′-AZA treatment and 38% and 35% in the 1 and 10 μM 5′-AZA treatment groups, respectively. Thus, it is assumed that 5-Aza-2′-deoxycytidine may improve ER by upregulating the expression of HOXA10 [53].

Maltseva L. I et al. (2019) studied the efficacy of epigallocatechin-3-gallate (EGCG) as a treatment for CE in women with reproductive impairment. The study included 38 women: 30 patients diagnosed with infertility and 8 with recurrent pregnancy loss. All patients were divided into 2 groups. The first group (15 patients) received EGCG at a dose of 400–600 mg/day b.i.d. for 2 months as part of complex therapy for CE. The control group included 23 patients who received complex, standard CE treatment [37]. The level of promoter methylation of the HOXA10 and HOXA11 genes was determined by RT-PCR with preliminary bisulfite conversion. Gene methylation analysis showed that 84% of biopsy samples (*n* = 32) demonstrated hypermethylation of the HOXA10 gene, 97% (*n* = 37) showed HOXA11 hypermethylation, and in the vast majority of cases—in 76.3% (*n* = 29) patients—a combination of both was identified [37]. After therapy with EGCG, HOXA10 and HOXA11 gene activity normalized in 93.4% (*n* = 14) and 83.4% (*n* = 11) of all cases, respectively. In the control group, demethylation of HOXA10 genes was observed in 17.3% (*n* = 4) women (OR: Odds ratio 66.5; 95% CI 6.68–66.16), HOXA11 activity normalized in 13% (*n* = 3) (OR: 18.3; 95% CI: 3.45–97.19), and two patients with primary infertility had higher levels of HOXA11 methylation after a course of standard therapy compared to baseline. Thus, the authors conclude that standard therapy is not capable of epigenetic regulation of the activity of the receptivity-related HOXA10 and HOXA11 genes in the vast majority of cases, whereas EGCG suppresses abnormal methylation, i.e., acts as an inhibitor of DNA methyltransferase, resulting in demethylation and reactivation of methylated silent genes [37].

Many studies have focused on the mechanisms of the biological activity of EGCG. There is an opinion that its action is based on its ability to inhibit inflammatory reactions that are common in diseases with a proliferative component [113,114]. According to certain authors, EGCG can inhibit the activation of NF-kB, a universal transcription factor that controls the expression of genes involved in the immune response, apoptosis, and cell cycle [115]. In addition, EGCG has a preventive effect against precancerous and cancerous conditions of the cervix by regulating abnormal epigenetic modifications [116], in particular, it suppresses abnormal DNA methylation, being a direct inhibitor of the DNMT enzyme [114].

Another agent with demethylating activity is indole-3-carbinol (I3C). It can regulate gene expression by modulating DNA methyltransferases, histone deacetylases, microRNAs, and several transcription factors such as aryl hydrocarbon receptor (AhR) and NF-kB and thus exerting its epigenetic demethylation effect [117]. A study in a model of pancreatic tumor cells using the PCR method, which allows for determining the level of DNA methylation, showed that I3C demethylated p16 INK4a from the gene promoter region in a dose-dependent manner and, as a result, reactivated it [118]. The use of I3C has been studied as part of a complex therapy in women with benign breast disease (fibrocystic dysplasia) and breast cancer with a BRCA1 mutation [119,120,121]. There are reports of the clinical use of I3C in the complex treatment of dysfunctional uterine bleeding and functional ovarian cysts [122,123].

Tayukina I.P. et al. (2010) studied the effect of epigenetic therapy with I3C on the morphology and function of the endometrium and its receptor status in various pathological conditions in patients with infertility [124]. The study included 70 women of reproductive age. Three groups were formed: Group Ia for simple typical endometrial hyperplasia (26%), Group Ib for CE (26%), Ic for endometrial hyperplasia in combination with CE (21%); the control group included women with normal endometrium (27%). The patients were treated with an I3C product at a dose of 300 mg/day for 2 months, then the dose of indole-3-carbinol was reduced to 100 mg/day. Treatment with indole-3-carbinol proved effective in restoring the implantation capacity of the endometrium and can be recommended to patients in ART programs [124].

Thus, a promising direction in the treatment of women with chronic diseases of the reproductive system, accompanied by infertility, miscarriage, and menstrual dysfunction, is the use of epigenetic drugs: EGCG and I3C, which inhibit DNA methyltransferases, thereby contributing to the normalization of the activity of the HOXA10 and HOXA11 genes and the morphofunctional state of the endometrium and the infertility treatment efficacy. The pregnancy rate with the use of EGCG and I3C in women with infertility and miscarriage is significantly higher compared to women receiving standard therapy. It is obvious that epigenetic therapy in the treatment of inflammatory and proliferative diseases of the uterus has great prospects for increasing the effectiveness of ART programs [37,117,124].

In recent years, epigenetic therapies for common diseases (primarily oncological), aimed at molecular intervention in their pathogenesis, have reached a new level of development, enabling the possibility of targeted epigenetic editing [125,126,127]. This progress has been largely driven by tremendous advances in molecular biology, genetics, and biochemistry, as well as the active implementation of cutting-edge high-throughput whole-genome sequencing and DNA microarray approaches in experimental and clinical research.

An important direction in modern epigenetic therapy is episensitization—the enhancement of sensitivity in resistant malignant tumors to standard chemotherapy, targeted therapy, and immunotherapy using epigenetic drugs [128]. Further prospects in this field are opened by the combined application of epigenetic therapy and the integration of novel “multi-omics” technologies [129].

While the presented data appear promising, they have limitations related to sample size, study design, and reproducibility. Further research is needed to obtain more conclusive evidence.

## 7. Discussion and Conclusions

The effectiveness of ART programs has stopped at 40% and we do not see further growth. Professionals have learned to work effectively with embryos, but at the same time, such an important property of the endometrium as receptivity is not given due attention. The development of reliable biomarkers for detecting ER defects may be a promising tool for increasing the number of successful implantations in ART programs. The analysis of the literature showed that abnormal hypermethylation of the promoter regions of the HOXA10 and HOXA11 genes is observed in women of reproductive age suffering from infertility due to CE, uterine fibroids, PCOS, and tuboperitoneal factor infertility. The methylation status of the HOXA10 and HOXA11 genes may be considered a potential predictor as part of the diagnosis and monitoring in the course of infertility treatment.

Current diagnostic systems allowing for the identification of ER defects (ERA^®^, nirsERT, rsERT) are expensive and not always available for monitoring infertility. An available method for assessing the methylation status of the HOXA10 and HOXA11 genes is real-time PCR with preliminary bisulfite conversion. Perhaps the development and implementation of such test systems into routine clinical practice will make it possible to optimize the solution to this problem.

We suppose that a promising drug therapy for improving ER is the combination of epigenetic agents epigallocatechin-3-gallate and indole-3-carbinol, which promote reactivation of the HOXA10 and HOXA11 genes and increase endometrial receptivity.

It should be noted that at present, most completed studies on the topic of epigenetic regulation of endometrial receptivity have been conducted in a limited number of patients, and the listed diagnostic and treatment methods aimed at improving the ART outcomes require further study.

## Figures and Tables

**Table 1:** Comparison of ERA, rsERT, and PCR-based DNA methylation tests.

Diagnostic Method	Brief Description/Biomaterial	Advantages	Disadvantages
ERA (Endometrial Receptivity Array) (Transcriptomic Analysis)	Analysis of 238+ gene expression to determine endometrial receptivity status and personalize the embryo transfer day (pWOI). Biomaterial: Endometrial biopsy.	Commercially available. Validated and widely used in clinical practice. Possesses the largest evidence base among molecular tests.	High cost. Invasive (pipelle biopsy). Requires an additional embryo transfer cycle. Insufficient validation in the general population. Contradictory data in patients with RIF.
rsERT (nirsERT) (Non-Invasive Transcriptomic Test)	RNA sequencing to assess the transcriptomic profile. Biomaterial: Uterine fluid.	Non-invasive (can be performed on a uterine fluid sample without biopsy). Can be conducted in the same cycle as embryo transfer.	High cost. Insufficient validation and clinical testing. Technical difficulties in sample collection/processing, risk of RNA degradation. Research method, not yet routine practice.
Tests for Gene Methylation Levels (HOXA10, HOXA11) (Epigenetic Analysis)	Assessment of methylation levels in gene promoter regions using PCR. Biomaterial: Endometrial biopsy.	Low cost. High specificity. Utilizes the widely available PCR method. Potential for rapid screening of many samples. Highly informative about endometrial functional state. Correlates with infertility duration and pathologies (chronic endometritis, endometriosis). Relative accessibility and low cost of the PCR method.	Invasiveness (biopsy). Limited validation and testing. Validation for other biomaterial types requires additional research.

## Data Availability

All data are open source, accessible and available from the corresponding author.
